# Estrogen deficiency is associated with hippocampal morphological remodeling of early postmenopausal mice

**DOI:** 10.18632/oncotarget.15702

**Published:** 2017-02-25

**Authors:** Yan Yan, Liang Cheng, Xin Chen, Qin Wang, Mingjing Duan, Jichao Ma, Linjing Zhao, Xuemei Jiang, Jing Ai

**Affiliations:** ^1^ Department of Pharmacology, Harbin Medical University, The State-Province Key Laboratories of Biomedicine-Pharmaceutics of China, Heilongjiang Province, Harbin 150081, China; ^2^ College of Bioinformatics Science and Technology, Harbin Medical University, Heilongjiang Province, Harbin 150081, China

**Keywords:** estrogen, postmenopause, morphology, transmission electron microscopy, hippocampus

## Abstract

Estrogen (E_2_) deficiency is reported to involve in the impairment of cognition in postmenopausal women. However, the morphological basis is still unclear. In the present study, using transmission electron microscopy (TEM), we observed the ultrastructure of hippocampus in female C57BL/6 mice at the age of 18 months (18 M) which is considered as the early stage of postmenopause (n = 8). Compared with control mice aged 6 M (n = 8), we identified that the morphological changes in the hippocampus of these menopausal mice were mitochondrial damage, lipofuscin deposition and microtubule degradation. Notably, after E_2_ was subcutaneously injected into mice aged 16 M with a dosage of 3.5 μg/kg every three days for two months in the 18 M + E_2_ group (n = 8), mitochondrial damage and lipofuscin deposition in the DG region of hippocampus were prevented, but the degraded microtubules in the hippocampus of postmenopausal mice were failed to restore. These data suggest that hippocampal ultrastructure remodeling in mice can be initiated at the early stage of postmenopause, E_2_ supplementation could only have an effect on mitochondrial damage and lipofuscin increase.

## INTRODUCTION

With the increasing of elderly population in the world, more and more individuals are suffering from senile dementia. It has been estimated that 115.4 million people globally will develop this cognitive disorder by 2050 [1]. Understanding the pathological mechanism of dementia could help us find new biomarker and yield novel therapeutic strategy. Previous studies have found that age-related loss of sex-steroid hormone can increase the risk of dementia, and women after menopause have greater risk to suffer from Alzheimer's disease (AD) as compared to age-matched men [2, 3], suggesting that estrogen may play a key role in the pathological development of dementia. Even though estrogen therapy has been applied to improve the cognitive function of postmenopausal women, the effects of this hormone replacement therapy (HRT) on dementia remain controversial in clinical trials [4, 5, 6, 7]. Instead of benefiting the cerebral functions, HRT was found detrimental to women aged 65 or older [5, 6], while no apparent negative effect was found in women aged from 50 to 59 [8]. This disparity of therapeutic effects may due to the difficulty for HRT to diminish the established pathological changes in patients more than 65 years old [9, 10, 11]. Overall, these phenomena suggest that initiating estrogen therapy at the early years of postmenopause (before ages 50 to 60 years) would in all probability be better than that at the late stage of postmenopause (at ages of more than 65 to 79 years). This also indicates that replenishing the insufficient estrogen level at the beginning of postmenopause may be the optimum timing to prevent cognitive impairment of women in the following years, which contributes to a new concept about HRT for the prevention of dementia. However, in terms of this early-initiated HRT, changes in the brain of postmenopausal women are largely unknown. Exploring the pathophysiological alterations of brain at the early stage of postmenopause and identifying the effects of estrogen (E_2_) in this process will greatly improve our understandings of HRT for the prevention of cognitive impairment in women.

Fortunately, many studies have been implemented to investigate the action of E_2_ in brain. Based on studies in postmenopausal female rodents, E_2_ was found to regulate the permeability of blood-brain barrier (BBB) [12] and improve the spatial reference memory evaluated by both morris water maze (MWM) [13, 14] and radial arm maze [15, 16, 17]. Besides that, E_2_ was also found to regulate synapse plasticity by increasing the expression of hippocampal SNAP25 and synaptophysin [18, 19, 20], and stimulate neurogenesis in dentate gyrus by activating estrogen receptors [21, 22, 23]. Moreover, the expression of neurotrophin like BDNF [24, 25] and the activity of NOS [26] could also be promoted by E_2_. E_2_ has been found to modulate glia-mediated neuronal inflammation pathway in aged female mice as well [27]. Experiments in aged monkeys revealed that E_2_ administration could restore the cognitive function of these non-human primates [28]. In addition, E_2_ was confirmed to improve the synaptic function [29] and exert the antioxidative effects [30, 31] in ovariectomized rats. It has the ability to inhibit the activity of apoptosis signaling [32] and increase the expression of anti-apoptosis gene *Bcl2* [33]. Furthermore, in hippocampal neurons *in vitro*, it has been documented that E_2_ can induce spinogenesis [34] and ameliorate excitotoxicity, oxidative injury, and amyloid beta-peptide toxicity [35]. These studies fundamentally evidenced that E_2_ played a pivotal role in dementia. However, the morphological changes of the brain regarding E_2_ deficiency are extremely limited, especially in the initiation of menopause. In this study, by transmission electron microscopy (TEM), we observed the ultrastructure of the hippocampus in mice at the early stage of postmenopause with and without E_2_ supplementation. The aim was to provide basic morphological evidences for early-initiated HRT.

## RESULTS

### Estrogen could prevent mitochondrial damage in the hippocampal DG region of early postmenopausal mice

**Figure 1:**
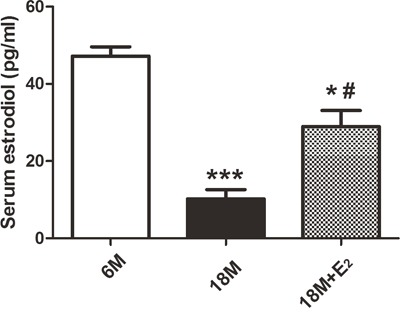
Estradiol concentration in mice serum The concentration of serum estradiol (pg/ml) was decreased in 18 M group in comparison with 6 M group (*n* = 8) (* *p* < 0.05, *** *p* < 0.001). Estradiol administration in 18 M + E_2_ group partly reversed the low level of estradiol in 18 M mice (*n* = 8) (# *p* < 0.05, unpaired *t*-test). Data are presented as mean ± SEM.

Firstly, serum estrogen levels in 6 M group, 18 M group and 18 M + E_2_ group were tested. As illustrated in Figure 1, serum estrogen levels in 18 M group (10.21±2.43 pg/ml) are less than those in 6 M group (47.22±2.39 pg/ml), and the E_2_ levels in 18 M + E_2_ group (28.97±4.16 pg/ml) indicate that E_2_ replacement effectively improves the decreased estrogen level in 18 M group.

**Figure 2:**
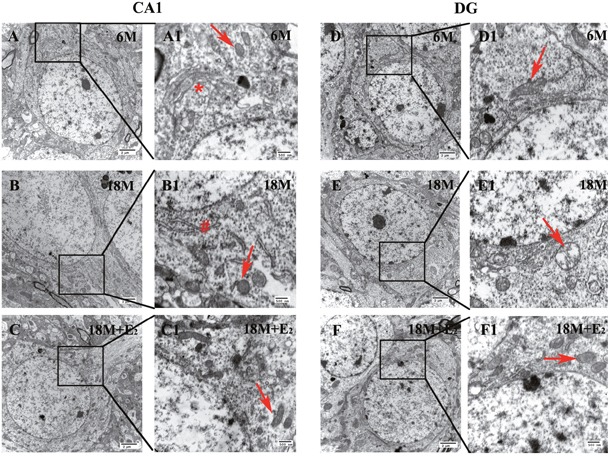
The morphology and ultrastructure of neurons in both CA1 and DG regions **A-F**. Pictures were taken by TEM at 10000 × magnification, neurons presented integrated nuclear membrane and normal chromatin distribution in both CA1 and DG regions of 6 M (A, D), 18 M (B, E) and 18 M + E_2_ (C, F) group. **A1-F1**. Certain fields of A-F were further observed at 30000 × magnification. **A1**. Organelles in the CA1 neuron of 6 M group were well-organized, which is represented by Golgi body (signed by asterisk). **B1**. In the CA1 neuron of 18 M group, healthy endoplasmic reticulum (signed by well number) and mitochondrion (signed by arrow) were detected. **C1**. In the CA1 neuron of 18 M + E_2_ group, intact mitochondria with high electron density could be seen. **D1**. Organelles in the DG neuron of 6 M group were prominent. **E1**. The mitochondria in the DG neuron of 18 M group were swelling with disappeared cristae. **F1**. All the organelles were well developed in the DG neuron of 18 M + E_2_ group. Scale bar = 2 μm in A-F and 500 nm in A1-F1.

Next, we used Transmission Electron Microscope (TEM) to observe the morphological changes of hippocampus. Compared with 6 M group (Figure 2A and 2D), the form of neuron and neuronal chromatin remained unchanged in both hippocampal CA1 (Figure 2B) and DG (Figure 2E) regions of 18 M group, and supplementation of E_2_ for 2 months did not affect the status as shown in 18 M + E_2_ group (Figure 2C and 2F). Importantly, the neuronal polarity was also preserved in CA1 subfield (Figure 2A-2C). Organelles including mitochondria, Golgi body and rough endoplasmic reticulum were all well developed in hippocampal CA1 region of experimental groups (Figure 2A1-2C1). In DG region, though Golgi body and rough endoplasmic reticulum presented no difference among 6 M, 18 M and 18 M + E_2_ groups, we found marked swelling of mitochondria coupled with disappeared cristae in 18 M group (Figure 2E1), which could be prevented by E_2_ administration (Figure 2F1).

### Estrogen ameliorated lipofuscin deposition in hippocampal DG neurons of early postmenopausal mice

**Figure 3:**
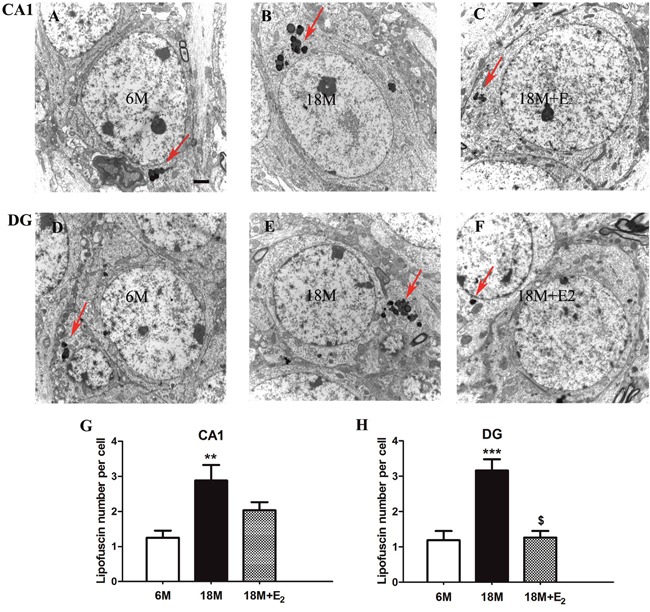
Lipofuscin deposition in the hippocampal neuron of mice **A**. Single lipofuscin presented in the CA1 subfield of 6 M group. **B**. A pile of lipofuscins were accumulated in the perinuclear region of CA1 neurons in 18 M group. **C**. The amount of lipofuscins in the CA1 neuron of 18 M + E_2_ group was limited. **D**. Only small amount of lipofuscins could be seen in the DG subfield of 6 M group. **E**. Great amount of lipofuscins was found in the DG neuron of 18 M group. **F**. It is hard to trace lipofuscin in the DG neuron of 18 M + E_2_ group. **G**. The number of deposited lipofuscins was remarkably increased in the CA1 region of 18 M group. **H**. Estrogen replacement in 18 M + E_2_ group can mitigate the deposition of lipofuscin in the DG region of 18 M group. Lipofuscin is composed by a group of spherical structures with varying degrees of electron density which is pointed by rigid arrow in each image. Scale bar = 2 μm. ** *p* < 0.01, *** *p* < 0.001 compared to 6 M group; $ *p* < 0.05 compared to 18 M group. Data are presented as mean ± SEM.

Lipofuscin is composed of a pile of spherical structures with varying degrees of electron density, it is often deposited in perinuclear region of cytoplasm [40]. In comparison with the limited amount of lipofuscins in both CA1 and DG regions of 6 M group (Figure 3A and 3D), we found marked accumulation of lipofuscins in relative regions of 18 M group, as indicated by arrows in Figure 3B and 3E. Furthermore, after elderly mice were treated with E_2_, lipofuscin accumulation was difficult to find in the DG region of 18 M + E_2_ group (Figure 3F) while not CA1 region (Figure 3C). Then the number of deposited lipofuscins in each group was calculated, and the results were shown in Figure 3G and 3H. The number of lipofuscins in each neuron of CA1 region was increased from 1.25±0.91 in 6 M group to 2.88±1.83 in 18 M group (*P* < 0.01). And in DG region, the number of lipofuscins in each neuron was increased from 1.07±1.05 in 6 M group to 3.17±1.79 in 18 M group (*P* < 0.001). Importantly, with the administration of E_2_, the accumulated lipofuscin number per neuron in DG region was significantly decreased to 1.28±1.01 (*P* < 0.001), but the number was 2.03±1.24 in CA1 region, which was not statistically different compared with that in 18 M group.

### Estrogen had no effect on the damaged microtubules of early postmenopausal mice

**Figure 4:**
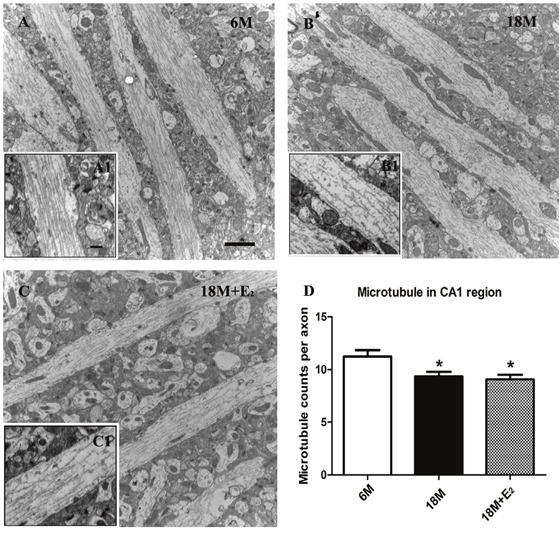
Estrogen was unable to ameliorate the destabilized microtubule in postmenopause mice **A**. Regular and integrated microtubules were detected in CA1 region of 6 M group; **A1**. Well-organized fiber-like microtubules were visible in the enlarged region. **B**. The neurons of 18 M mice displayed inconsecutive microtubules; **B1**. Great intervals between microtubules in axon of neuron could be seen. **C**. Microtubules in 18 M + E_2_ mice also presented a less-organized morphology; **C1**. The continuity and amount of microtubules declined in neurons. **D**. Compared with 6 M group, the mean number of axonal microtubules in 18 M group was greatly decreased as well as in 18 M + E_2_ group. (* *p* < 0.05 compared to 6 M group). Data are expressed as mean ± SEM. Scale bar = 2 μm in A-C and 500 nm in A1-C1.

Microtubules play a key role in transporting neurotransmitters to the synapse, and they act as downstream effectors of neurotransmitter signaling in the target neuron [41]. Therefore, the morphology of hippocampal microtubule was detected in this study. As shown in Figure 4A and 4A1, neuronal microtubules were filled in axons with relative continuous and fiber-like texture in 6 M group. However, discontinuous and sparse microtubule arrays were observed in axons of 18 M group (Figure 4B and 4B1). To our surprise, the supplementation of E_2_ to postmenopausal mice failed to prevent the damage of microtubules (Figure 4C and 4C1). The statistical data in Figure 4D further manifested that the number of microtubules was significantly decreased in 18 M group compared with 6 M group, and E_2_ administration in 18 M + E_2_ group did not quantitatively reverse the defective microtubules of elderly mice.

### Estrogen had no effect on the unchanged synapses of early postmenopausal mice

**Figure 5:**
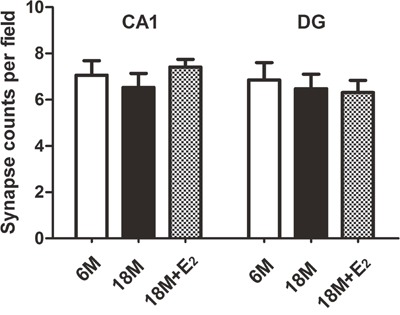
Synapse density in the hippocampus of mice In hippocampal CA1 and DG subfields, the synapse number per field has no statistical difference among 6 M, 18 M and 18 M + E_2_ groups. (*n* = 22, one-way ANOVA). Data are presented as mean ± SEM.

**Figure 6:**
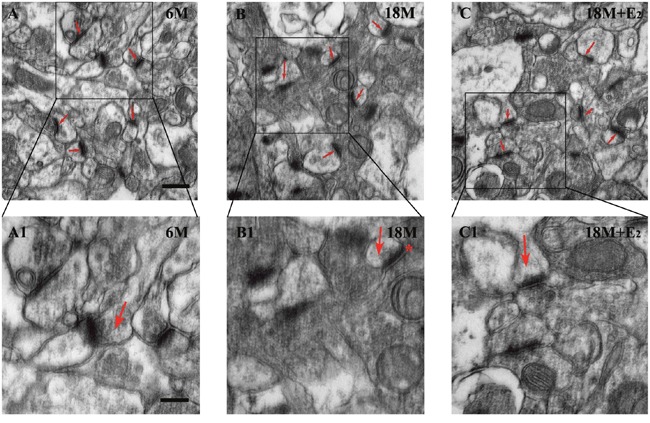
Estrogen had no effect on the morphology of synapse in postmenopause mice **A**. Typical synapse including presynaptic and postsynaptic regions as well as synaptic cleft was observed in hippocampal CA1 neuron of 6 M mice; **A1**. Presynaptic vesicles were detected in the regional magnified version. **B**. In 18 M group, synapses were also prominent in morphology; **B1**. The synaptic cleft was clearly recognized and marked by an asterisk. **C**. The morphological appearance of synapse in 18 M + E_2_ mice was comparative with the other two groups; **C1**. Active zone thickness, synaptic cleft width and the amount of presynaptic vesicles were similar to the other groups. Active zone of synapse is arrowed in A-C. Arrows in A1-C1 point to the presynaptic region. Scale bar = 500 nm in A-C and 250 nm in A1-C1.

Previous studies have reported that estrogen can regulate synapse plasticity [18, 19], so we calculated the number of synapses in both CA1 and DG regions. Intriguingly, there was no difference in these two subfields among three groups, indicating that menopause could not lead to the loss of synapses (Figure 5). In mice of 6 M group, many round vesicles containing neurotransmitters were filled in the presynaptic region. Likewise, these phenomena were found in both 18 M and 18 M + E_2_ groups. Moreover, no difference in the amount of vesicles was observed among these three groups (Figure 6A-6C). To further observe the synapse, enlarged images were obtained. We found that the typical morphologic features of synapse including presynaptic dense band, relative thin postsynaptic membrane and synaptic cleft were all similar among these three groups (Figure 6A1-6C1).

## DISCUSSION

In the present study, we illustrated a comprehensive picture of morphological alterations in the hippocampus of early postmenopausal mice, and evaluated the role of E_2_ in these mice by TEM technique. We concluded that the main features of structure remodeling in the hippocampus of early postmenopausal mice were mitochondrial damage, lipofuscin deposition and microtubule degradation. Furthermore, we identified that E_2_ supplementation could prevent the onset of the mitochondrial damage and lipofuscin deposition rather than microtubule degradation caused by menopause. These findings suggest that hippocampal structure remodeling can be initiated at the early stage of postmenopause, but not all of the alterations can be reversed by E_2_ supplementation. Thus, the application of HRT in early postmenopausal women (before ages 50 to 60 years) cannot be too optimistic based on our data. To some extent, we also provide the morphological evidences for the failure of HRT in clinical trials of late postmenopausal women (at ages of more than 65 to 79 years).

Mitochondrial dysfunction is a common feature of both aging and neurodegenerative diseases [42, 43, 44]. Improvement of mitochondrial function is considered as a strategy to slow aging and aging-related neurodegenerative disorders [45, 46]. In this study, we found that abnormal mitochondrial structure was elicited in DG but not CA1 region of hippocampus at the early stage of postmenopause, and E_2_ supplementation could prevent the changes in DG region, indicating that E_2_ may be useful to preserve the structure of mitochondria in this early phase. The protective effect of E_2_ is probably exerted through its action in oxidative stress as previously reported [47, 48, 49]. The results also indicate that neurons in DG region are easier to be affected by E_2_ deficiency than neurons in CA1 region.

Interestingly, except for the abnormal mitochondria, the morphologies of organelles including Golgi apparatus, endoplasmic reticulum as well as cell membrane remained unchanged in the hippocampal neuron of 18 M group, suggesting that mitochondrial damage is prior to the injury of other organelles at the early stage of postmenopause. This is in line with a previous report [50]. Clinical data reported that, in women aged 65 and older, HRT negatively influenced verbal memory and brain volume [5], and HRT was ineffective for mild to moderate AD in women aged from 56 to 91 [51] as it missed the best time to reverse the established pathological changes. Here, we first demonstrated that developed mitochondrial structural remodeling may be the partial fundamental evidence of HRT resistance for the cognitive impairment treatment at both early and late stages of postmenopause.

As a marker of aging, lipofuscin has been reported to deposit in different areas of brain in models like 24 M female hamsters and rats [52, 53, 54, 55]. Lipofuscin is the intralysosomal electron-dense remnants filled by oxidized proteins and lipid clusters that cannot be digested by lysosome [56, 57]. The existence of lipofuscin is considered as the response to oxidative stress [58]. Mitochondrion is the key organelle responding to oxidative stress, the dysfunction of it will lead to the production of a large amount of reactive oxygen species [59, 60]. Hence, the deposition of lipofuscin is considered as the outcome of mitochondrial damage [61]. In this study, the number of lipofuscins was significantly increased in both CA1 and DG regions of hippocampus in 18 M mice, and E_2_ supply could effectively prevent the lipofuscin deposition in DG region. This result is consistent with changes of mitochondria, suggesting that the deposited lipofuscin might be associated with the damage of mitochondria. Of interest, it has been documented that E_2_ administration could reduce lipofuscin deposition in neurons of 12 M, 18 M and 24 M female rats [53, 62], but it failed to benefit the impaired cognitive ability of OVX rats aged 17-18 M [63]. These phenomena imply that the functional benefit of E_2_ is not simply associated with the decreased lipofuscin deposition.

Moreover, we observed the morphology of both neuronal microtubule and synapse of the hippocampus, due to their involvement in neurotransmission. We found that, though decreased number of microtubules was observed in the hippocampal neurons of 18 M mice, which is consistent with data from aging people [64, 65], the change was failed to be prevented by E_2_ supplementation. This result suggests that microtubule damage in mice at the early stage of postmenopause is not originated from E_2_ deficiency. During the process of estrogen supplementation in this study, the mice also went through the process of aging. Hence, some pathophysiological changes in aging may be involved in the damage of microtubules, including the increase of ROS and the decrease of neurotrophic factors [66, 67]. However, to figure out the mechanism of microtubule damage, further exploration is needed. Additionally, some literatures have reported that the expression of SNAP25 and synaptophysin, two components of soluble N-ethylmaleimide-sensitive factor attachment protein receptor (SNARE), decreased in hippocampus of 18-24 M rats [20] and 27-28 M mice [13]. Since SNARE is an engine that mediates the fusion of vesicular and presynaptic membranes [68], the dysfunction of it may result in the accumulation of vesicles in presynapse. In the cortex of aging monkeys with low level of estrogen, accumulated vesicles in axon terminals were also reported [69]. While, in our work, no synaptic vesicle accumulation was detected in the hippocampus of both solvent-injected and E_2_-administrated 18 M mice.

The synapse density along with the active zone thickness and synaptic cleft width were also similar among groups in this study, which is in line with evidences from mouse [70], rat [71], monkey [72] and human being [73]. Nevertheless, the reduced number of synapse in aging rats like 30 M female Fisher 344 rats has also been published [74, 75, 76]. This discrepancy may due to the different ages of the animals. For example, the disturbance of neurotransmitter release in brain was detected only when the rats were over 18 M [77, 78]. In the present study, mice at the age of 18 M were detected, in which the degeneration of synapse may not exhibit yet. Therefore, the unchanged synapse density in our experimental mice is reasonable, based on the hints from a previous study which mentioned that aging rats with good memory tend to have the same synapse density in the DG region of hippocuampus like young rats [79].

Taken together, although E_2_ supplementation could prevent menopause-elicited mitochondrial damage and lipofuscin deposition, it failed to block the defective microtubules. These findings provide an experimental evidence of the unapparent effect of HRT in the early phase of postmenopause women and give a possible explanation for HRT failure at the late stage of postmenopause.

## MATERIALS AND METHODS

### Animals

Female C57BL/6 mice used in the experiments were separated into three groups: mice at the age of 6 months (6 M) without any injection were used as control 6 M group (*n* = 8), while mice at the age of 18 months (18 M) without and with the supplementation of E_2_ were designed as 18 M group (*n* = 8) and 18 M + E_2_ group (*n* = 8), respectively. The animals were raised at 23 ± 1°C and maintained on 12 h dark-light artificial cycle (lights on at 07:00 A.M.) with food and water available *ad libium*. All animal procedures were approved by the ethic committees of Harbin Medical University and the Institute of Laboratory Animal Science of China (A5655-01). Moreover, the protocols complied with the guidance for the Care and Use of Laboratory Animals of the US National Institutes of Health (NIH Publication No. 85–23, revised 1996).

### Estrogen (E_2_) administration

Since the reproductive function of mice declines with time as the estrous cyclicity becomes acylic from 11 M to 16 M [80, 81], E_2_ administration was started in mice at the age of 16 M and lasted for two months, aiming to mimic a replenishment for the decreased E_2_ in early postmenopausal phase. Therefore, in the 18 M + E_2_ group, E_2_ (17β-estradiol, Sigma, E-2758, St Louis, MO, USA) dissolved in peanut oil was subcutaneously injected into mice with a dosage of 3.5 μg/kg every three days for two months. The injection was also performed in 18 M group, but only solvent was injected.

### Mouse estradiol ELISA

At the age of 18 M, mice were anesthetized (pentobarbital sodium, 60 mg/kg, i.p.) before the blood was extracted from the left ventricle and maintained at room temperature for 3 h, the serum was collected after centrifugation (3000 rpm, 5 min), and stored at -80°C. Similarly, serum was obtained from 6 M mice. The concentration of estradiol in serum was measured using the mouse/rat estradiol ELISA kit (Calbiotech, ES180S-100, Spring Valley, CA) following the manufacturer's instructions.

### Transmission electron microscopy

Based on stereotaxic coordinates of hippocampus, coronal slices of mouse brain were prepared and soaked immediately in 2.5% glutaraldehyde. After 6-8 h at 4°C, they were cut into 1mm thick coronal slices and the hippocampi part was separated. Next, samples were rinsed with PBS (0.1 M) before post-fixed by osmium tetroxide for 1-2 h. The dehydration procedure was carried out using ethanol and acetone in concentration gradients. Subsequently, we used epoxy resin for embedding prior to the slicing of ultra-thin sections. Then, double staining by uranium acetate and lead citrate was performed. And finally, images were taken by transmission electron microscope (JEM-1220, JEOL Ltd., Tokyo, Japan).

### Statistical analysis

The numbers of lipofuscin, synapse and microtubule were calculated in a blinded manner. And the amount of microtubules per axon was obtained by averaging three transverse sections. Data are expressed as mean ± SEM. GraphPad Prism 5.0 was used for statistical analysis. For the comparison among groups, one-way ANOVA was applied and *P* < 0.05 was considered as statistical significance.
